# Supplementary material for: Macrophage-derived IGF-1 protects the neonatal intestine against necrotizing enterocolitis by promoting microvascular development
Source: Commun Biol. 2022 Apr 6;5:320. doi: 10.1038/s42003-022-03252-9 (PMC8987083; doi:10.1038/s42003-022-03252-9)
Supplement: Supplementary file 2 — Supplementary Information [file 42003_2022_3252_MOESM2_ESM.pdf]

Supplementary Information for

Macrophage-derived IGF-1 protects the neonatal intestine against necrotizing enterocolitis by promoting microvascular development

Xiaocai Yan[1,2], Elizabeth Managlia[1,2], You-Yang Zhao[3,4], Xiao-Di Tan[2,5] and Isabelle G. De Plaen[1,2]*

S.R. Isabelle G. De Plaen
Email: isabelledp@northwestern.edu

This PDF file includes:
Figures S1 to S4.

Supplemental Figure 1

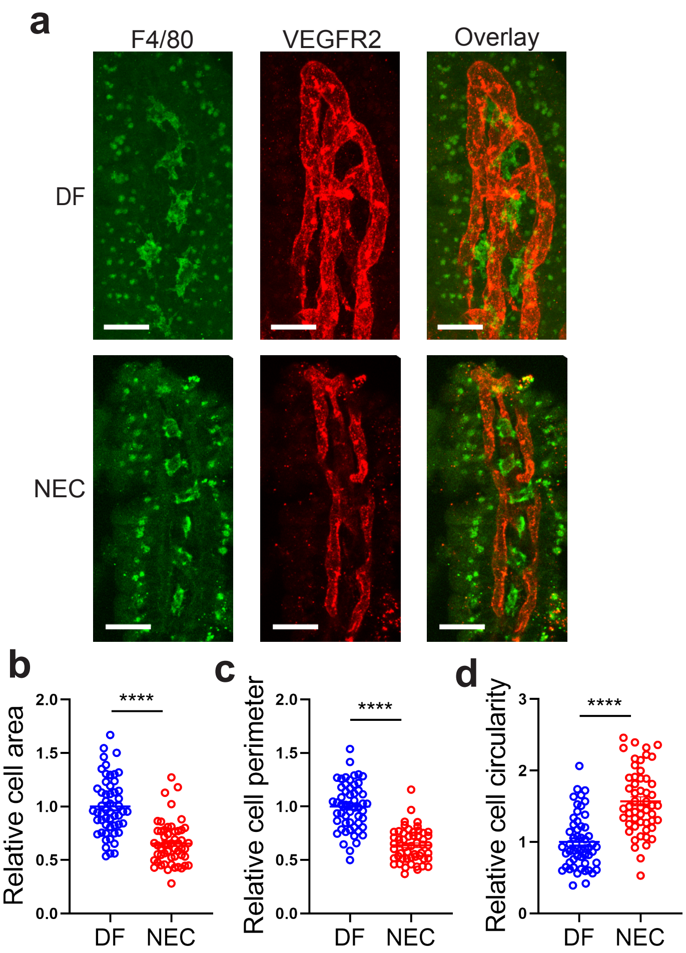

Supplemental Fig. 1 Intestinal macrophage morphology changes during NEC development. Whole mount intestinal tissues from 24h NEC and DF littermates were stained with F4/80 and VEGFR2. (**a**) Representative images are shown. Relative F4/80$^+$ cell area (**b**), perimeter (**c**), and circularity (**d**) of more than 50 villous F4/80$^+$ cells in each group were measured by Lasso and measurement tool of Photoshop software. Scale bar=25μm. n=6 pups in each group. *P* values were calculated using 2-sided Student's *t* tests (**b-d,** results expressed as mean ± SEM). ****p<0.0001. Source data are provided as a Source Data file.

Supplemental Figure 2

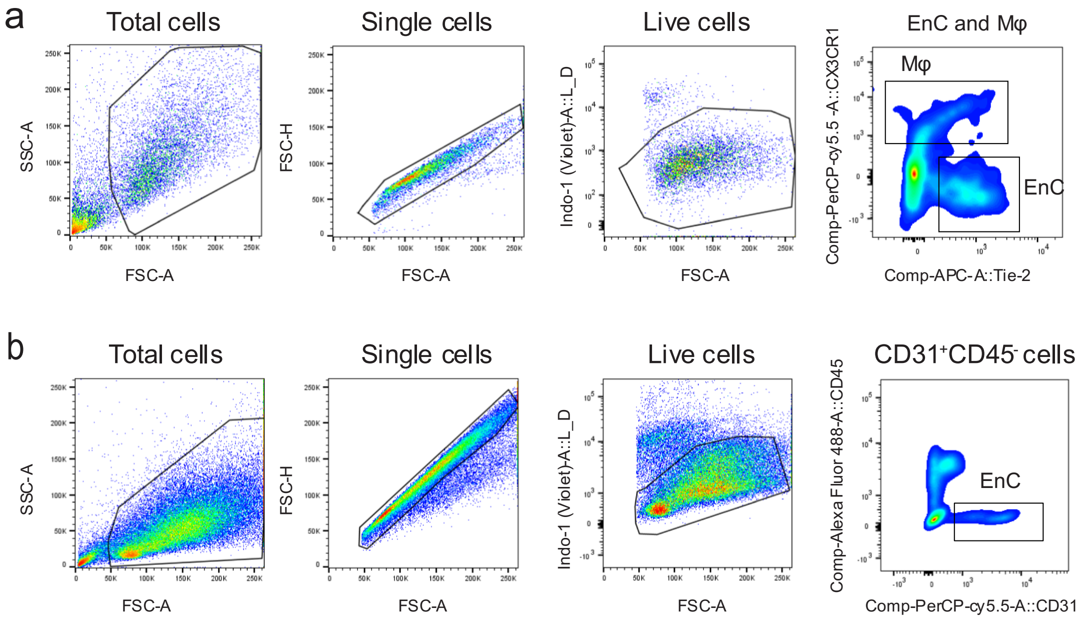

Supplemental Fig. 2. **a** Gating strategy for Figure 3d and 3g**:** $5x10^4$ intestinal CX3CR1$^+$ macrophages and $10x10^4$ endothelial cells enriched by magnetic column selection were cultured separately or together in 24-well plates. Cells were collected at 48 hours of culture and stained with live/dead blue, anti-Tie-2, CX3CR1, Ki-67, IGF-1 antibodies, or isotype control for flow cytometric analysis. To assess endothelial cell proliferation (Figure 3d), and endothelial cell and macrophage IGF-1 expression (Figure 3g), collected cells gated on single, live were further gated on Tie-2$^+$ to identify endothelial cells (EnC) or on CX3CR1$^+$ cells to identify macrophages (Mφ) for further analysis. **b** Gating strategy for Figure 5f and Figure 7a: To assess endothelial cell proliferation (Figure 5f and figure 7a), isolated small intestinal LP cells were stained with live/dead blue, anti-CD45, CD31, Ki-67 antibodies and gated on single, live, CD31$^+$CD45$^-$ to identify endothelial cells (EnC).

Supplemental Figure 3

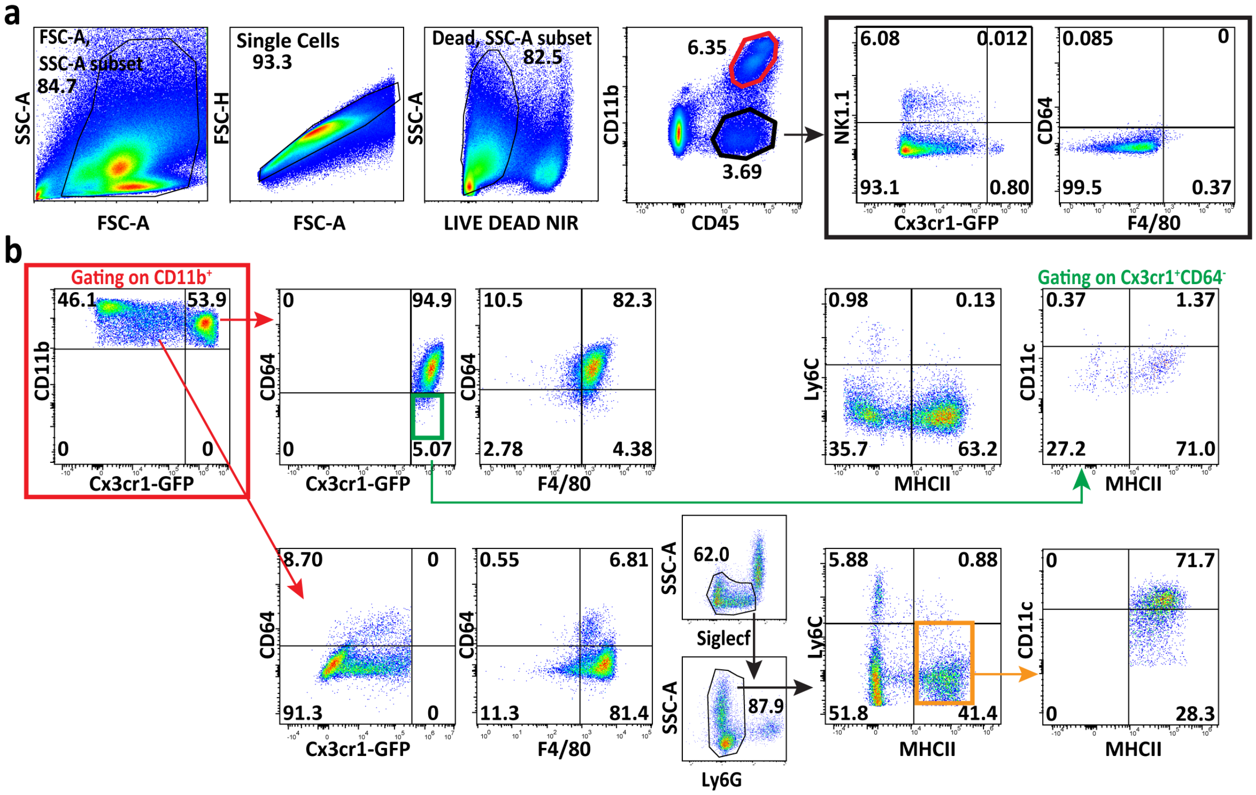

Supplemental Fig. 3 Intestinal lamina propria CX3CR1$^+$ cells express CD11b, F4/80, CD64, but were negative for Ly6C, CD11c and NK1.1: Lamina propria cells were obtained from CX3CR1-GFP expressing pups and stained for CD45, CD11b, NK1.1, F4/80, Ly6C, CD64, CD11c, Siglec F, Ly6G and MHCII. **a** Neonatal mouse intestinal lamina propria live, single, CD45$^+$CD11b$^-$ cells were gated (black gate and box) and analyzed for GFP (CX3CR1) and the NK cell marker NK1.1 or F4/80 and CD64. The vast majority of the neonatal intestinal NK cells were CX3CR1$^-$. **b** To evaluate the specificity of CX3CR1 as a macrophage marker, live, single, CD45$^+$CD11b$^+$ cells were gated (red gate and box) and analyzed for GFP (CX3CR1) expression. GFP$^+$ (top b panel) or GFP$^-$ (bottom b panel) cells were then assessed for CD64 and CX3CR1 or for CD64 and F4/80 expression. The majority of CX3CR1$^+$ cells co-expressed CD64 and F4/80 while the CX3CR1$^-$ cells contained only rare CD64$^+$ cells. Monocytes were examined in the GFP$^+$ (top b panel) population by analyzing Ly6C and MHCII. Dendritic cells in the GFP$^+$ population were examined by first gating the CD64$^-$ cells (green gate) followed by assessment of CD11c and MHCII expression. Only rare dendritic cells and monocytes were present in the GFP$^+$ population. For monocyte analysis of the GFP$^-$ population (bottom b panel), cells were analyzed for Ly6C and MHCII expression after gating out eosinophils (Siglecf) and neutrophils (Ly6G). Dendritic cells in the GFP$^-$ population were analyzed based on CD11c and MHCII of the Ly6C$^-$ MHCII$^+$ cells (orange gate). Dendritic cells and monocytes were mainly present in the GFP$^-$ population.

Supplemental Figure 4

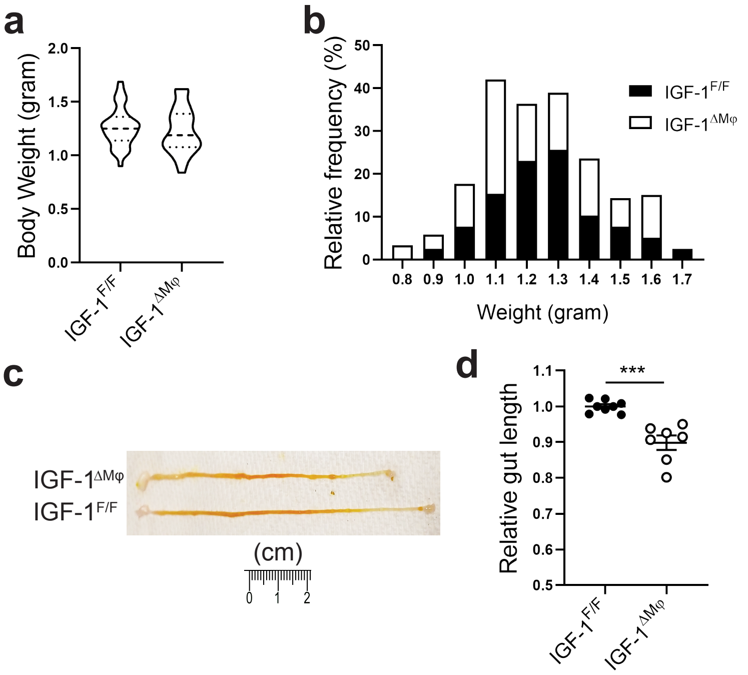

Supplemental Fig. 4 Neonatal pups with IGF-1-deficient macrophages weigh less and have shorter intestine compared to controls. a-b Body weight (violin plot, **a**) and weight distribution (bar graph, **b**) of *Igf-1*$^{\Delta M\phi}$ (*n*=30) and IGF-1-sufficient control littermates (*Igf-1*$^{f/f}$, *n*=39) are shown. **c-d** Intestines obtained from four litters of 1 to 4-day-old *Igf-1*$^{f/f}$ (*n*=8) and *Igf-1*$^{\Delta M\phi}$ pups (littermates with similar weight, *n*=7) were dissected and measured from the stomach to the cecum: **c** Representative image of the intestine of 24 hour-old *Igf-1*$^{f/f}$ and *Igf-1*$^{\Delta M\phi}$ pups; **d** Intestinal length was compared between the two groups. *P* values were calculated using 2-sided Student's *t* tests (**a** and **d,** results expressed as mean ± SEM). ***p<0.001. Source data are provided as a Source Data file.
