# Supplementary material for: Macrophage-derived IGF-1 protects the neonatal intestine against necrotizing enterocolitis by promoting microvascular development
Source: Commun Biol. 2022 Apr 6;5:320. doi: 10.1038/s42003-022-03252-9 (PMC8987083; doi:10.1038/s42003-022-03252-9)
Supplement: Supplementary file 3 — Description of Additional Supplementary Files [file 42003_2022_3252_MOESM3_ESM.pdf]

Description of Additional Supplemental Files

File name: Supplementary Data 1

Description: Source Data for Main Figures.
