# Supplementary material for: Macrophage-derived IGF-1 protects the neonatal intestine against necrotizing enterocolitis by promoting microvascular development
Source: Commun Biol. 2022 Apr 6;5:320. doi: 10.1038/s42003-022-03252-9 (PMC8987083; doi:10.1038/s42003-022-03252-9)
Supplement: Supplementary file 5 — Reporting Summary [file 42003_2022_3252_MOESM5_ESM.pdf]

nature portfolio

Corresponding author(s): Isabelle G. De Plaen

Last updated by author(s): 02-10-2022

Reporting Summary

Nature Portfolio wishes to improve the reproducibility of the work that we publish. This form provides structure for consistency and transparency in reporting. For further information on Nature Portfolio policies, see our Editorial Policies and the Editorial Policy Checklist.

Statistics

For all statistical analyses, confirm that the following items are present in the figure legend, table legend, main text, or Methods section.

n/a	Confirmed	
☐	☒	The exact sample size (*n*) for each experimental group/condition, given as a discrete number and unit of measurement
☐	☒	A statement on whether measurements were taken from distinct samples or whether the same sample was measured repeatedly
☐	☒	The statistical test(s) used AND whether they are one- or two-sided<br>*Only common tests should be described solely by name; describe more complex techniques in the Methods section.*
☒	☐	A description of all covariates tested
☐	☒	A description of any assumptions or corrections, such as tests of normality and adjustment for multiple comparisons
☐	☒	A full description of the statistical parameters including central tendency (e.g. means) or other basic estimates (e.g. regression coefficient) AND variation (e.g. standard deviation) or associated estimates of uncertainty (e.g. confidence intervals)
☐	☒	For null hypothesis testing, the test statistic (e.g. *F*, *t*, *r*) with confidence intervals, effect sizes, degrees of freedom and *P* value noted<br>*Give P values as exact values whenever suitable.*
☒	☐	For Bayesian analysis, information on the choice of priors and Markov chain Monte Carlo settings
☒	☐	For hierarchical and complex designs, identification of the appropriate level for tests and full reporting of outcomes
☒	☐	Estimates of effect sizes (e.g. Cohen's *d*, Pearson's *r*), indicating how they were calculated

Our web collection on statistics for biologists contains articles on many of the points above.

Software and code

Policy information about availability of computer code

Data collection	No specific software was used.
Data analysis	The statistical software GraphPad Prism (version 8.1.2) was used for statistical analysis

For manuscripts utilizing custom algorithms or software that are central to the research but not yet described in published literature, software must be made available to editors and reviewers. We strongly encourage code deposition in a community repository (e.g. GitHub). See the Nature Portfolio guidelines for submitting code & software for further information.

Data

Policy information about availability of data

All manuscripts must include a data availability statement. This statement should provide the following information, where applicable:

- Accession codes, unique identifiers, or web links for publicly available datasets
- A description of any restrictions on data availability
- For clinical datasets or third party data, please ensure that the statement adheres to our policy

No novel datasets were generated in this study. Data that support the findings of this study are available from the corresponding author upon reasonable request. Gt(ROSA)26Sortm4(ACTB-tdTomato,-EGFP)Luo/J (mT/mG), B6.129P2(Cg)-Cx3cr1tm1Litt/J (Cx3cr1-GFP), B6J.B6N(Cg)-Cx3cr1tm1.1(cre)Jung/J (Cx3cr1-Cre), B6.129(FVB)-Igf1tm1Dlr/J (Igf1flox) were commercially available as described in the method section. Source data were provided.

Field-specific reporting

Please select the one below that is the best fit for your research. If you are not sure, read the appropriate sections before making your selection.

☒ Life sciences ☐ Behavioural & social sciences ☐ Ecological, evolutionary & environmental sciences

For a reference copy of the document with all sections, see nature.com/documents/nr-reporting-summary-flat.pdf

Life sciences study design

All studies must disclose on these points even when the disclosure is negative.

Sample size	Sample size have been defined for each experiment and is based on previously conducted power analysis for similar primary outcome.
Data exclusions	N/A. Pups from both sexes will be used, as no consistent association between sex and rates of NEC has been identified.
Replication	Experiments were run at least three times unless indicated otherwise. Appropriate negative and positive controls were used.
Randomization	Sex and Weight-matched littermates were randomly assigned to treatment or control group.
Blinding	For morphometric determination (e.g. histology score, sprout length, etc.) the investigator was blinded to the sample group assignment.

Reporting for specific materials, systems and methods

We require information from authors about some types of materials, experimental systems and methods used in many studies. Here, indicate whether each material, system or method listed is relevant to your study. If you are not sure if a list item applies to your research, read the appropriate section before selecting a response.

Materials & experimental systems

n/a	Involved in the study	
☐	☒	Antibodies
☒	☐	Eukaryotic cell lines
☒	☐	Palaeontology and archaeology
☐	☒	Animals and other organisms
☐	☒	Human research participants
☒	☐	Clinical data
☒	☐	Dual use research of concern

Methods

n/a	Involved in the study	
☒	☐	ChIP-seq
☐	☒	Flow cytometry
☒	☐	MRI-based neuroimaging

Antibodies

Antibodies used	The details of antibodies used have been described in the method section.
Validation	All antibodies were used according to manufacturer's recommendation.

Animals and other organisms

Policy information about studies involving animals; ARRIVE guidelines recommended for reporting animal research

Laboratory animals	Commercially available animals including C57BL/6, Gt(ROSA)26Sortm4(ACTB-tdTomato,-EGFP)Luo/J (mT/mG), B6.129P2(Cg)-Cx3cr1tm1Litt/J (Cx3cr1-GFP), B6J.B6N(Cg)-Cx3cr1tm1.1(cre)Jung/J (Cx3cr1-Cre), B6.129(FVB)-Igf1tm1Dlr/J (Igf1flox) purchased from Jackson Laboratory (Bar Harbor, ME) and both sexes were used.
Wild animals	This study did not involve wild animals.
Field-collected samples	N/A
Ethics oversight	Northwestern IACUC number IS00010688.

Note that full information on the approval of the study protocol must also be provided in the manuscript.

Human research participants

Policy information about studies involving human research participants

Population characteristics	Neonatal infants (less than 6 months of age) of both sexes undergoing intestinal tissue resection for NEC or for other pathologies.
Recruitment	Neonatal infants (less than 6 months of age) of both sexes undergoing intestinal tissue resection for NEC or for other pathologies were enrolled. All legal guardians of the participants signed informed consent forms.
Ethics oversight	The study was approved by the Lurie Children's Hospital of Chicago Institutional Review Board.

Note that full information on the approval of the study protocol must also be provided in the manuscript.

Flow Cytometry

Plots

Confirm that:

☒ The axis labels state the marker and fluorochrome used (e.g. CD4-FITC).

☒ The axis scales are clearly visible. Include numbers along axes only for bottom left plot of group (a 'group' is an analysis of identical markers).

☒ All plots are contour plots with outliers or pseudocolor plots.

☒ A numerical value for number of cells or percentage (with statistics) is provided.

Methodology

Sample preparation	Mouse intestinal tissues and intestinal lamina propria cells were used for study. The details of processing were described in the method section.
Instrument	LSRFortessa cell analyzer was used for data acquisition.
Software	FlowJo 10.7.1 was used for analysis.
Cell population abundance	N/A.
Gating strategy	Cells were gated on live, singlet cells for indicated markers analysis.

☐ Tick this box to confirm that a figure exemplifying the gating strategy is provided in the Supplementary Information.
